# Power to mechanical power to minimize ventilator-induced lung injury?

**DOI:** 10.1186/s40635-019-0243-4

**Published:** 2019-07-25

**Authors:** Pedro Leme Silva, Lorenzo Ball, Patricia R. M. Rocco, Paolo Pelosi

**Affiliations:** 1 Laboratory of Pulmonary Investigation, Centro de Ciências da Saúde, Carlos Chagas Filho Institute of Biophysics, Federal University of Rio de Janeiro, Av. Carlos Chagas Filho, s/n, Bloco G-014, Ilha do Fundão, Rio de Janeiro, RJ 21941-902 Brazil; 2 Department of Surgical Sciences and Integrated Diagnostics (DISC), University of Genoa, Largo Rosanna Benzi, 8, 16131 Genoa, Italy; 3 IRCCS AOU San Martino, Largo Rosanna Benzi, 10, 16132 Genoa, Italy

**Keywords:** Mechanics, Ventilator-induced lung injury, Mechanical energy, Mechanical power

## Abstract

Mechanical ventilation is a life-supportive therapy, but can also promote damage to pulmonary structures, such as epithelial and endothelial cells and the extracellular matrix, in a process referred to as ventilator-induced lung injury (VILI). Recently, the degree of VILI has been related to the amount of energy transferred from the mechanical ventilator to the respiratory system within a given timeframe, the so-called mechanical power. During controlled mechanical ventilation, mechanical power is composed of parameters set by the clinician at the bedside—such as tidal volume (*V*_T_), airway pressure (Paw), inspiratory airflow (*V*′), respiratory rate (RR), and positive end-expiratory pressure (PEEP) level—plus several patient-dependent variables, such as peak, plateau, and driving pressures. Different mathematical equations are available to calculate mechanical power, from pressure-volume (PV) curves to more complex formulas which consider both dynamic (kinetic) and static (potential) components; simpler methods mainly consider the dynamic component. Experimental studies have reported that, even at low levels of mechanical power, increasing *V*_T_ causes lung damage. Mechanical power should be normalized to the amount of ventilated pulmonary surface; the ratio of mechanical power to the alveolar area exposed to energy delivery is called “intensity.” Recognizing that mechanical power may reflect a conjunction of parameters which may predispose to VILI is an important step toward optimizing mechanical ventilation in critically ill patients. However, further studies are needed to clarify how mechanical power should be taken into account when choosing ventilator settings.

## Background

Mechanical ventilation is a supportive therapy used to maintain respiratory function and reduce work of breathing during surgical intervention and in critically ill patients with and without acute respiratory distress syndrome [1, 2]. However, mechanical ventilation can itself damage the lungs, causing what is known as ventilator-induced lung injury (VILI); the severity of VILI depends on the ventilator settings [3]. Some factors are directly set on the ventilator by the clinician, such as tidal volume (*V*_T_), driving pressure (Δ*P*), airflow (*V*′), respiratory rate (RR), and positive end-expiratory pressure (PEEP) [2]. Others depend on the patient’s respiratory system or on the patient-ventilator interaction, such as peak and plateau pressures, as well as Δ*P*.

VILI represents the unwanted result of a complex interplay among various mechanical forces, which act on lung structures, such as type I and II epithelial cells, endothelial cells, macrophages, peripheral airways, and extracellular matrix (ECM), during mechanical ventilation [1, 2]. The main mechanisms that can lead to VILI are direct damage to the alveolar capillary membrane and ECM, and mechanotransduction, which is the conversion of a mechanical stimulus into intracellular biochemical and molecular signals. The degree of both direct damage and mechanotransduction may depend on the amount of power transferred from the mechanical ventilator to the patient’s lungs. In turn, the degree of power transfer depends on ventilatory parameters adjusted by the operator at bedside [2].

## What is energy transfer from the respiratory muscles or mechanical ventilator to the patient’s lungs?

The energy expended to move the lungs from their resting position (i.e., functional residual capacity) to a given point of the pressure-volume (PV) curve can be provided by muscle contractions generating muscular pressure, as observed during spontaneous breathing, or artificially by a mechanical ventilator that generates airway pressure (Paw). Two of the first studies which calculated mechanical energy were done in infants with bronchopulmonary dysplasia, by measuring the area under the PV curves during spontaneous breathing [4, 5]. One demonstrated a positive association between increase in mechanical energy and increased lung elastance and airway resistance [4], while in the second, infusion of methylxanthines and diuretics [5] was followed by a decrement in mechanical energy. In physics, mechanical energy is the sum of potential and kinetic energies. This theoretical concept is applicable to respiratory physiology. In this context, mechanical energy depends on the position where the inspiratory effort starts within the respiratory system PV curve and on the driving force exerted by the respiratory muscles to generate chest wall movement. In the last 3 years, the concept of mechanical energy gained new attention from the critical care community when it was recognized that ventilator parameters can interact with forces acting on the lung surface and contribute to VILI [3, 6–8]. The mechanical ventilator can replace, partially or completely, the effort done by the respiratory muscles, but at the cost of increased airway pressures. These, in turn, must overcome the elastic and resistive forces of the respiratory system to generate movement (kinetic energy fraction), whereas the static component (potential energy) is reflected by the PEEP level, which in fact represents the baseline tension of the respiratory system (assuming a relaxed system without muscle activity).

## How is mechanical power calculated?

**Fig. 1:**
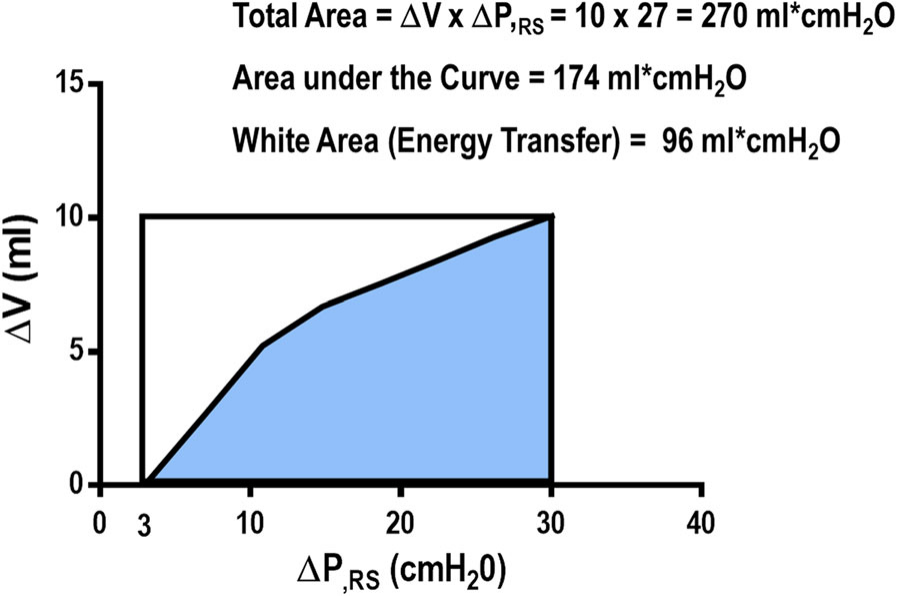
Experimental quasi-static PV curve from 3 to 30 cmH_2_O performed with a flexiVent® mechanical ventilator (SCIREQ, Montreal, QC, Canada). The total area, obtained by multiplying the volume difference (Δ*V*) by the pressure difference (Δ*P*_RS_) during the maneuver, was determined (270 mL cmH_2_O). Then, the area under the PV curve was calculated (174 mL cmH_2_O) and subtracted from the total area, yielding the white area (96 mL cmH_2_O)

The amount of energy transferred from the ventilator to the patient is measured in joules (J), while power is defined as the amount of energy transferred per unit of time (J/min). There are at least three different ways to calculate mechanical power (energy per breath times respiratory rate) with different degrees of complexity. The first method is based on an analysis of quasi-static PV curves of the respiratory system. Estimation of mechanical power with this method is largely dependent on the technique used to perform the PV curve. Under low-flow conditions, the influence of the resistive properties will be reduced, and the elastic properties of the respiratory system will be the main component of the mechanical energy calculation. Figure 1 shows an experimental quasi-static PV curve from 3 to 30 cmH_2_O performed with a flexiVent® mechanical ventilator (SCIREQ, Montreal, QC, Canada). The total area of the rectangle, obtained by multiplying the volume difference (ΔV) by the pressure difference (Δ*P*_RS_) was determined [270 mL cmH_2_O or 26.5 mJ]. The area under the curve was then calculated as the integral of the pressure with respect to volume (174 mL cmH_2_O or 17.1 mJ) and subtracted from the total area of the rectangle, yielding the white area, which corresponds to energy transfer (96 mL cmH_2_O or 9.4 mJ). To convert from mL cmH_2_O to joules, all variables should be transformed to SI units, in which 1 mL would correspond to 10^−6^ m^3^, while 1 cmH_2_O would correspond to 98.1 Pa. As Pa m^3^ = J, 1 cmH_2_O mL would correspond to 98.1 × 10^−6^ Pa m^3^ or 98.1 × 10^−3^ mJ. This value (energy), multiplied by the respiratory rate, gives power. Using this method, the potential energy generating static strain in the respiratory system (PEEP) is not considered.

**Fig. 2:**
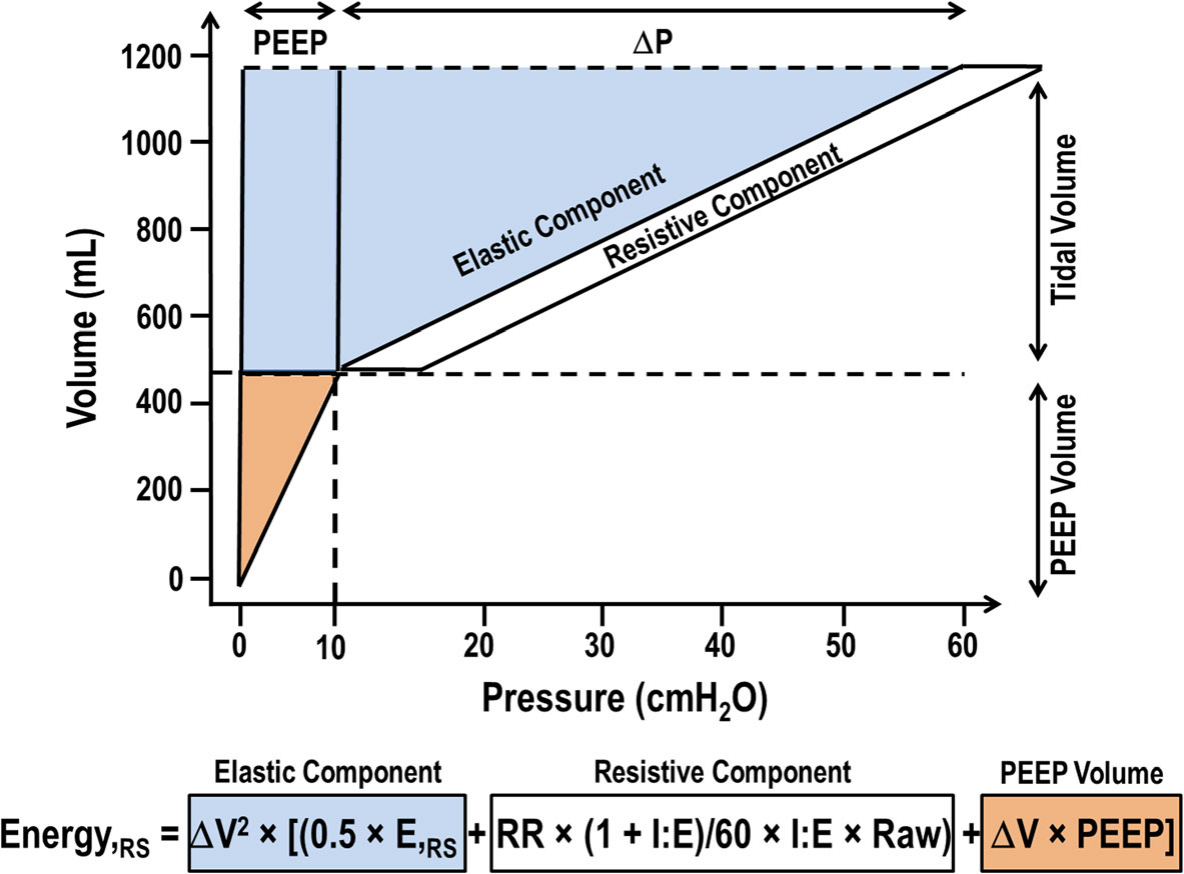
Mechanical power calculation, which includes the resistive properties and variation of lung volume correspondent to PEEP level. All components are depicted below the schematic figure: elastic, resistive, and PEEP volume

In the second method, the calculation of mechanical power includes both the resistive properties (endotracheal tube/airways and tissue resistance) and the variation of lung volume correspondent to the PEEP level (Fig. 2) [3, 6]:

$$ \mathrm{Power}{,}_{\mathrm{RS}}=0.098\times \mathrm{RR}\times \left\{\Delta {V}^2\times \left[\left(0.5\times E{,}_{\mathrm{RS}}+\mathrm{RR}\times \left(1+\mathrm{I}:\mathrm{E}\right)/60\times \mathrm{I}:\mathrm{E}\times \mathrm{Raw}\right)+\Delta V\times \mathrm{PEEP}\right]\right\} $$

The major advantage, according to the authors who developed this mathematical description of mechanical power, is that it enables the quantification of the relative contribution of its different components (*V*_T_, RR, Δ*P*_RS_, PEEP, I:E, airflow) and may predict the effects of their changes [3]. The partitioning of mechanical power components was done increasing one parameter while keeping the others constant. As pointed out by the authors themselves, the effects of each component on power are not always predictable in clinical practice, because in several conditions, changing one parameter will necessarily modify others (e.g., if *V*_T_ is reduced, RR is typically increased to maintain constant minute ventilation).

**Fig. 3:**
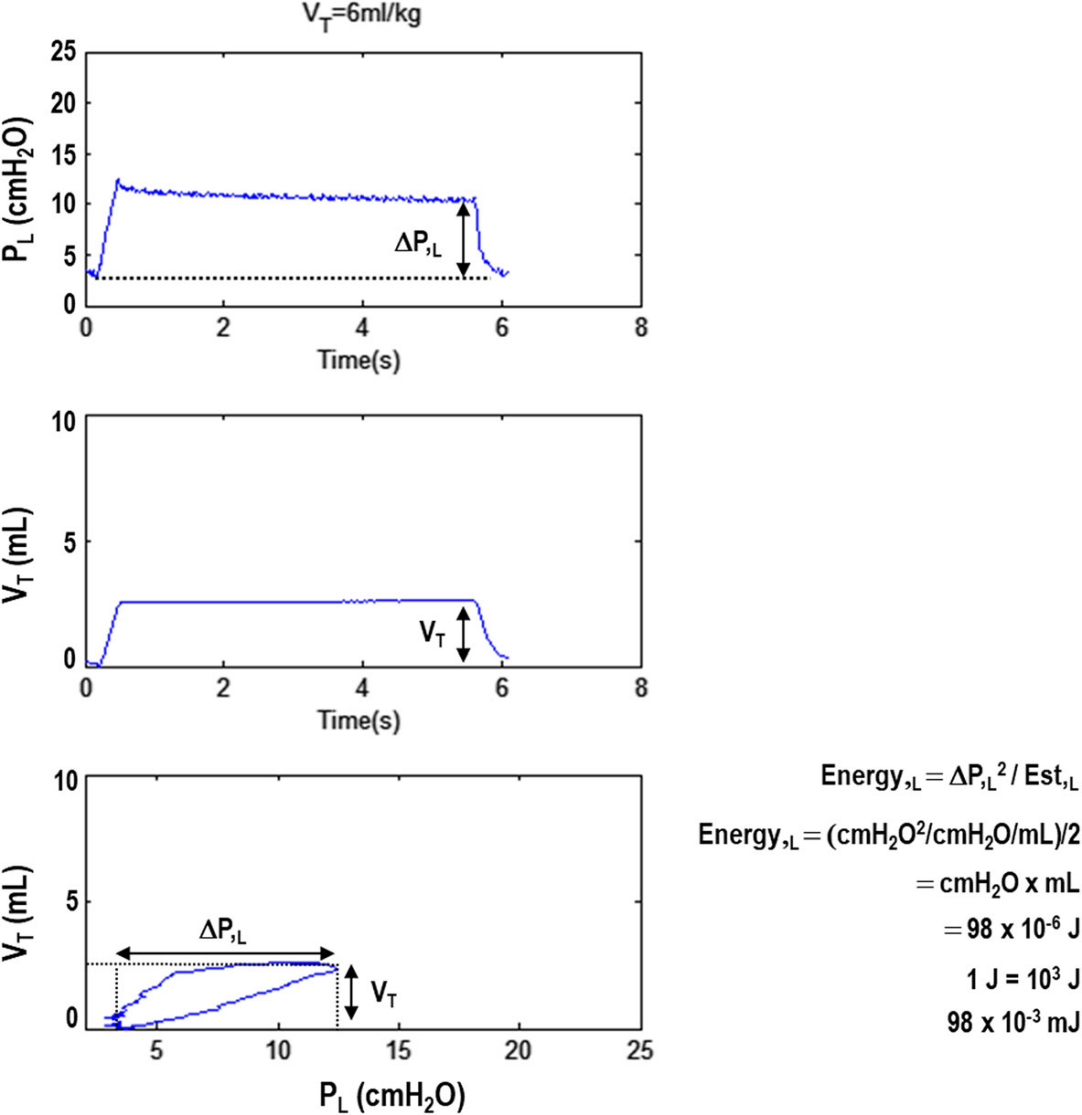
Representative curves of transpulmonary pressure (*P*,_L_), tidal volume, and volume-*P*,_L_ curve. *V*_T_, tidal volume; Δ*P*,_L_, transpulmonary driving pressure; Est,_L_, static lung elastance. Mechanical energy (Energy,_L_) was calculated based on the equation described by Guerin et al. [19] and the simplified formula of Marini and Jaber [9] as Energy,_L_ = Δ*P*,_L_^2^/Est,_L_ = Δ*P*,_L_^2^/(Δ*P*,_L_/*V*_T_) = Δ*P*,_L_ × *V*_T_, which is the area of the rectangle. Therefore, one must compute the area of the rectangle and divide the result by two. This simplified equation estimates elastic work without taking into account resistive properties and PEEP

The third method to calculate mechanical power is performed by implementing intra-tidal inspiratory pauses (Fig. 3). This calculation does not take into account the resistive component or PEEP level and has been considered a simplification of the second method mentioned above. This equation computes the most important component (driving mechanical power) [9], because although peak airway pressure has been shown to be important in experimental models, the excursion of tidal pressure seems to be more important [10]. The net effect of increasing PEEP level in terms of outcomes during mechanical ventilation may depend on its ability to increase the lung surface area, which will decrease the excursion of tidal pressure, and the mechanical energy transfer from ventilator to lung.

Several studies have computed the Δ*P*_RS_ instead of Δ*P*,_L_. However, Δ*P*_RS_ comprises the effects of both the lung and chest wall, as well as abdominal stiffness, which can be relevant in critically ill patients [11]. Δ*P*,_L_ was calculated by subtracting transpulmonary pressure at end-inspiration—i.e., the difference between the pressure in the alveoli and the pressure in the pleural cavity (chest wall)—and at end-expiration.

## Mechanical power and diseased lungs: what about normalization?

**Fig. 4:**
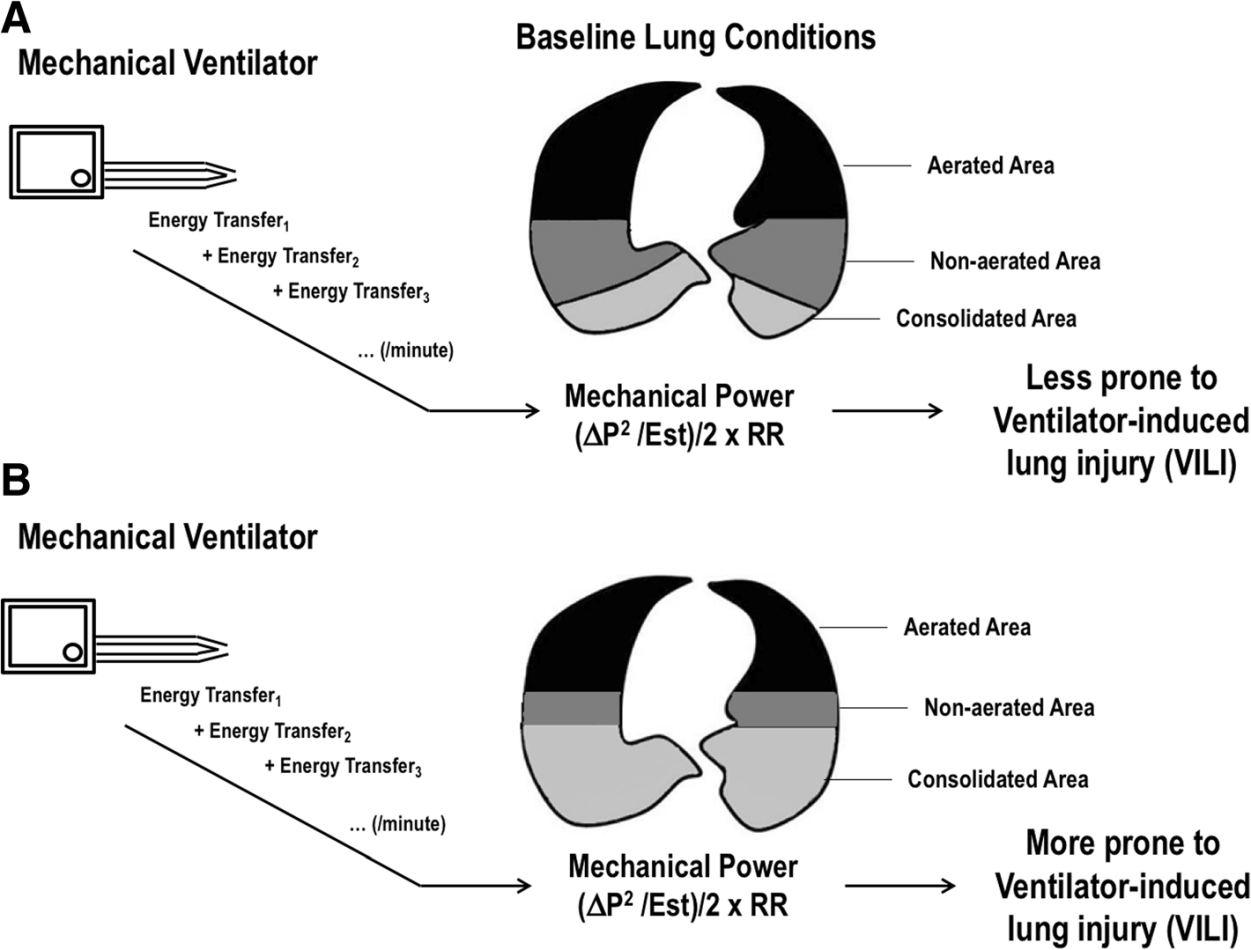
**a**, **b** Interaction of a given mechanical power with baseline lung conditions. If the lung surface (encompassing both aerated and non-aerated areas) able to accommodate the energy transfer is high, VILI is less likely to occur. On the other hand, if the lung surface is low, VILI is more likely to develop for the same mechanical power delivered by the mechanical ventilator

The major determinants of VILI are volutrauma and atelectrauma [2, 12]. By measuring the extent and distribution of inflammation with [18F]-fluorodeoxyglucose uptake in two experimental models of VILI, Güldner et al. showed that volutrauma causes greater inflammation than atelectrauma [13]. In a comment to this study, Tonetti et al. [14] used the average values to compute the mechanical power delivered to the respiratory system in the two groups. In the volutrauma group, the average mechanical power to the respiratory system was 17.12 J/min, higher than that computed in the atelectrauma group (7.13 J/min). The major parameter differing between volutrauma and atelectrauma groups was the PEEP level; *V*_T_ and RR were comparable. Nevertheless, whether mechanical power should be normalized considering the available lung surface which will absorb it is still a matter of debate. Although the question remains unanswered, some insights are available from post hoc analyses. The first derives from the fact that mechanical power can be normalized to the lung tissue mass available for ventilation; the nomenclature “intensity” was proposed for this parameter [13]. For a given mechanical power, intensity is higher in lungs with fewer ventilated areas, as well as at the interface between lung zones with different mechanical properties [15]. Figure 4 depicts the interaction of a given mechanical power with baseline lung conditions. If the lung surface able to accommodate the mechanical power transfer is large, VILI is less likely to occur. On the other hand, if the lung surface is small, VILI is more likely to develop for the same mechanical power delivered. Not only the lung surface is important to VILI progression, but also the open/closed interfaces, which have been associated with high [(18F)FDG] uptake, increasing proportionally to the severity of the lung condition [16]. Therefore, both the total area to be ventilated and the inhomogeneous poorly inflated or uninflated compartment represent important parameters to be monitored and used for normalization of mechanical power transfer. The damage threshold has yet to be defined for humans. In this line, in a secondary analysis of patients enrolled in two previously published randomized controlled trials, namely Acurasys [17] and Proseva [18], Guerin et al. [19] attempted to define a safe threshold for mechanical power. Using the most simple method to calculate mechanical power (*V*_T_ × Δ*P*_RS_ × RR), the authors found that mechanical power above 12 J/min was associated with reduced survival. This threshold was similar to that detected in a previous study done in large animals [6]. Recently, Serpa Neto et al. performed a post hoc analysis of data from 8207 critically ill patients admitted to the ICUs of 59 hospitals in the USA in order to examine the association between mechanical power and in-hospital mortality [20]. They found that high mechanical power was independently associated with higher mortality in ICU patients who received invasive ventilation for at least 48 h.

## As long as mechanical power is low, can I modify ventilator settings? Theory vs. practice

Each component of the mechanical power equation has its own weight for the final calculation. In an elegant previous study [3], the authors ran several computations by changing one variable at a time while keeping all others constant. They showed that when *V*_T_ was increased by 20%, mechanical power increased by 37%. In an experimental model of endotoxin-induced ARDS in rats, at low mechanical power, high *V*_T_ was associated with VILI [7]. The authors emphasized that control of *V*_T_ seems more important than control of RR. In this study, maintaining low mechanical power (~ 75 mJ/min) did not prevent lung damage when *V*_T_ was high (22 mL/kg). Additionally, multiple linear regressions were compared to the overall mechanical power. *V*_T_ predicted changes in IL-6 better than the pooled mechanical power construct did (*r*^2^ = 0.71 vs. 0.19, respectively), while for diffuse alveolar damage, their predictive capacities were comparable (*r*^2^ = 0.46 vs. 0.47). In other words, in a condition of low mechanical power, *V*_T_ should still be kept low, as it is itself capable of causing lung injury.

Mechanical power also increased by 37% when inspiratory airflow was increased by 20% [3]. In the previous experimental study done in large animals [6], mechanical power was increased from 2 to 22 J/min by increasing RR and inspiratory airflow. Increased RR at a similar *V*_T_ range has been associated with lung damage in experimental conditions [21] and with hemodynamic impairment [22]. Inspiratory airflow is closely associated with shear stress at the top of the cells within the respiratory bronchi. Some reports have associated inspiratory flow profiles with gas exchange, work of breathing, and cardiovascular functions [23–25]. Not only is inspiratory airflow associated with major physiologic consequences, expiratory flow is also an important indicator of changes in lung mechanics as acute lung injury progresses. Expiration is a passive process that uses elastic energy stored during inflation to drive airflow. If the potential energy stored after inspiration is low and not sufficient to return the system to a relaxed equilibrium before the next inspiration begins, flow continues throughout expiration and the alveolar pressure remains positive at end-expiration, exceeding the clinician-selected PEEP value [26]. In fact, this has been emphasized by a recent editorial [27] about how sudden deflation from a high airway pressure has the potential to trigger lung damage from a vascular point of view [28]. Under such conditions of high potential for high kinetic energy transfer, vascular flows and pressures are powerful determinants of VILI, especially in fragile, diseased lungs. This mechanism may contribute to lung heterogeneity, which may play an important role at the micro level [6]. Although no experimental studies have assessed modification of inspiratory or expiratory airflow while keeping mechanical power low, it is likely that alterations in airflow would induce lung damage.

According to a previous theoretical study, mechanical power was only increased by 5.7% when PEEP was increased by 20% [3]. The rationale to include PEEP as a component of mechanical power is that, at FRC, the lung is already partially stressed and strained; with PEEP application, there is an increase in lung volume corresponding to an increase in end-expiratory lung volume (EELV) and to an increased end-expiratory transpulmonary pressure, but in a static condition. This pressure is stored in lung structures as potential energy [29]. This fraction of static strain can be very prominent and may cause lung damage when compared to dynamic strain. It should be pointed out that lung inflation requires a further increase in transpulmonary pressure, which will reflect the dynamic strain related to the respiratory cycle. In most ICU ventilators, work-of-breathing computations do not include PEEP or EELV, since PV curves start from point (*x*, *y* = 0, 0). Although PEEP has less weight in mechanical power calculations than *V*_T_, airway pressure, or inspiratory airflow, the authors justify its presence in the mechanical power formula because it is associated with the potential fraction of mechanical energy, which by definition must be computed for total mechanical energy calculation. Nevertheless, the effect of PEEP level goes further than simply entering in the mechanical power calculation. By changing the EELV, PEEP has the ability to modify the lung surface area able to receive the stress released by the mechanical ventilator. In fact, the effect of mechanical power on respiratory system mechanics may depend on the recruitability of the patient’s lungs. If an increase in PEEP will lead to a decrease in driving pressure and respiratory system elastance, mechanical power will ultimately decrease, and vice-versa: if an increase in PEEP level fails to reduce or even increases driving pressure and respiratory system elastance, mechanical power will increase because of impairment of respiratory system mechanics due to overdistension of alveolar units.

**Fig. 5:**
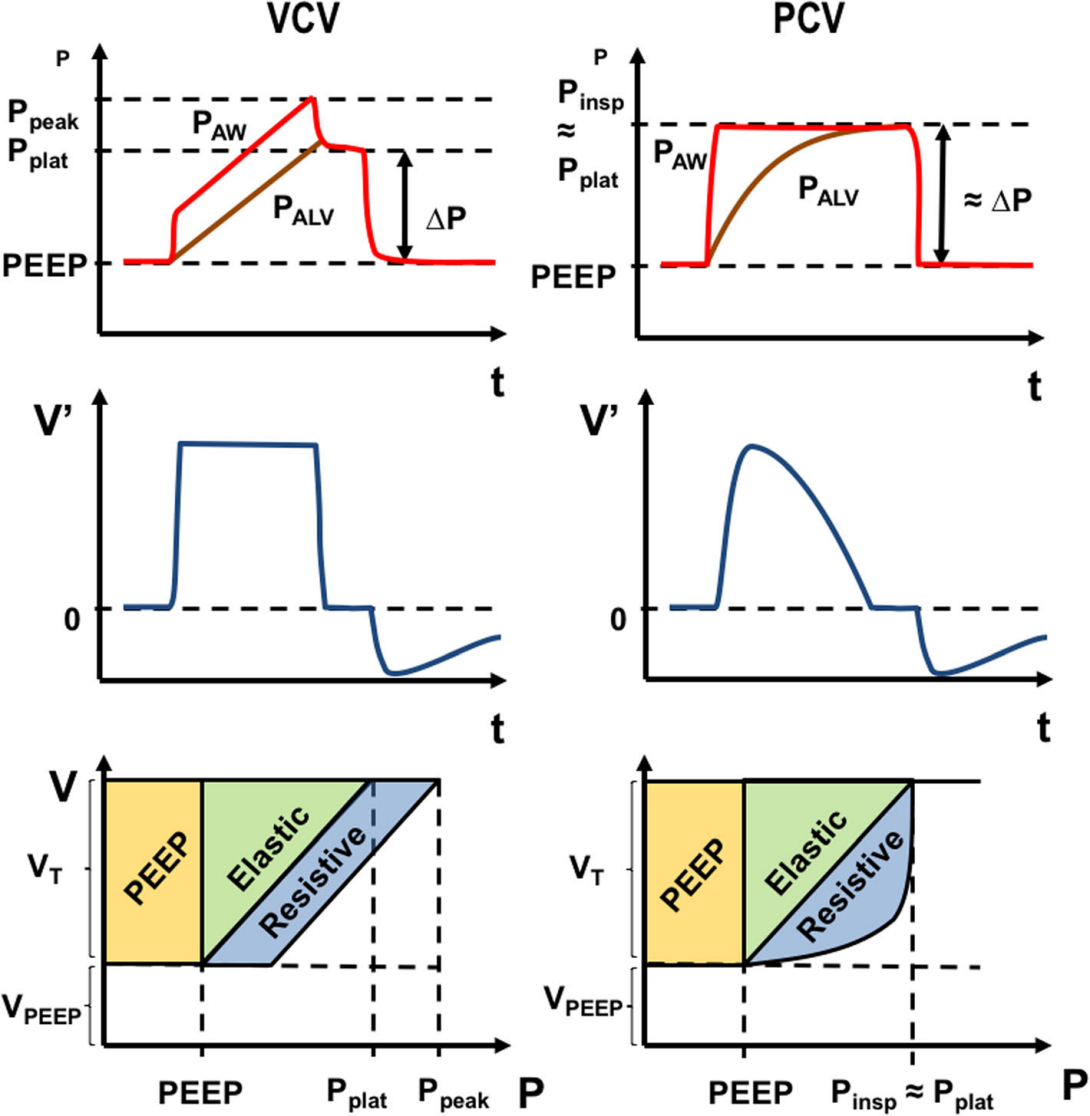
Mechanical power during volume- and pressure-controlled ventilation. Top panels represent the typical pressure-time curve of these two controlled ventilation modes; middle panels, the flow-time curves; and bottom panels, the weight of different power components in the two ventilation modes. VCV, volume-controlled ventilation; PCV, pressure-controlled ventilation

Mechanical power may also differ according to the mode of mechanical ventilation (pressure-controlled ventilation (PCV) or volume-controlled ventilation (VCV)), even when ventilator settings are the same. Mechanical power is probably higher in VCV than in PCV, as suggested by the illustrative diagram shown in Fig. 5: in PCV, peak pressure is equivalent to plateau pressure, while in VCV, peak pressure is higher than plateau pressure due to the resistive component. However, it is still unclear whether the resistive and elastic components of power have the same biological impact. In short, the type of ventilation should be taken into account when evaluating the effects of mechanical power on lung injury.

Current knowledge on the concept of mechanical power has limitations that should be addressed in future studies. First, all studies to date have focused on VCV. It would be of interest to investigate the effects of other ventilation modes, particularly pressure-controlled and assisted ventilation. During assisted ventilation, mechanical power is provided by the mechanical ventilator in tandem with the respiratory muscles [30]. New theoretical [31] and experimental [32] studies have dissociated the mechanical power imparted by the machine and that imparted by the respiratory muscles during assisted mechanical ventilation. However, further studies are needed to definitively determine mechanical power during assisted ventilation. Second, the relative weight and the interaction between different mechanical power components in real-world settings have yet to be determined. Finally, the microscopic mechanisms of energy transfer and the role of different anatomical areas of the lung and different cell lines remain unclear.

## Conclusions

The degree of lung damage in VILI can be linked to the amount of energy transferred from the mechanical ventilator to the respiratory system within a given timeframe, a construct known as mechanical power. There are several ways of calculating mechanical power, from simple formulas to highly complex equations. All have distinct benefits and shortcomings; some compute static mechanical energy and resistive pressure, while others disregard these parameters. Regardless of the way in which mechanical power is calculated, it is worth stressing that not all alveolar units will be exposed to it. Therefore, efforts should focus on normalizing mechanical power to the lung surface area amenable to ventilation. The recognition that mechanical power may reflect a conjunction of parameters which can predispose to VILI is an important step toward better care of critically ill patients.

## Abbreviations

*E*,_RS_: Respiratory system elastance
ECM: Extracellular matrix
EELV: End-expiratory lung volume
FRC: Functional residual capacity
I:E: Inspiratory/expiratory ratio
Paw: Airway pressure
PCV: Pressure-controlled ventilation
PEEP: Positive end-expiratory pressure
PV: Pressure-volume
Raw: Airway resistance
RR: Respiratory rate
*V*′: Airflow
VCV: Volume-controlled ventilation
VILI: Ventilator-induced lung injury
*V*_T_: Tidal volume
Δ*P*,_L_: Transpulmonary driving pressure
Δ*P*_RS_: Respiratory system driving pressure
Δ*V*: Delta volume
